# Pro-oxidant effect of α-tocopherol in patients with Type 2 Diabetes after an oral glucose tolerance test – a randomised controlled trial

**DOI:** 10.1186/1475-2840-6-8

**Published:** 2007-02-22

**Authors:** Mark S Winterbone, Mike J Sampson, Shikha Saha, Jackie C Hughes, David A Hughes

**Affiliations:** 1 Institute of Food Research, Norwich Research Park, Norwich, NR4 7UA, UK; 2 Bertram Diabetes Research Unit, Norfolk & Norwich University Hospital NHS Trust, Norwich, NR4 7UY, UK

## Abstract

**Background:**

As a part of a larger study investigating the effects of α-tocopherol on gene expression in type 2 diabetics we observed a pro-oxidant effect of α-tocopherol which we believe may be useful in interpreting outcomes of large intervention trials of α-tocopherol.

**Methods:**

19 type 2 diabetes subjects were randomised into two groups taking either 1200 IU/day of α-tocopherol or a matched placebo for 4 weeks. On day 0 and 29 of this study oxidative DNA damage was assessed in mononuclear cells from fasted blood samples and following a 2 h glucose tolerance test (GTT).

**Results:**

On day 0 there was no significant difference in oxidative DNA damage between the two groups or following a GTT. On day 29 there was no significant difference in oxidative DNA damage in fasted blood samples, however following a GTT there was a significant increase in oxidative DNA damage in the α-tocopherol treatment group.

**Conclusion:**

High dose supplementation with α-tocopherol primes mononuclear cells from patients with type 2 diabetes for a potentially damaging response to acute hyperglycaemia.

## Background

Type 2 diabetes is associated with an increased risk of atherosclerosis. Increased oxidative stress and damage to lipoproteins, cell membrane components and chromosomal DNA may play a role in this increased risk of atherosclerosis [1,2]. Increased susceptibility to oxidative DNA damage has been reported in type 2 diabetes [3,4], and we have shown recently an inverse relationship between oxidative DNA damage and telomere length in blood monocytes from patients with type 2 diabetes [5]. The potential role of oxidative stress in atherogenesis made antioxidant interventions appealing as a vascular risk reduction strategy, but there has subsequently been a lack of evidence of improved vascular outcomes in large scale antioxidant clinical trials [6,7]. A recent meta-analysis has also suggested an increased risk of all-cause mortality from vitamin E supplementation [8]. Potential reasons for this lack of benefit have been reviewed [9], as has the possible pro-oxidant effect of antioxidants in disease processes with existing high background levels of oxidative stress [10].

In this report we show an increase in oxidative DNA damage in mononuclear cells from patients with type 2 diabetes who had been supplemented with 1200 IU/d α-tocopherol, following a glucose tolerance test. This level of supplementation was chosen as we previously have shown no effect on DNA strand breaks or oxidisability with a lower dose of 400 IU α-tocopherol daily [3], but higher doses have shown a reduction in DNA single strand breaks using the comet assay [11].

## Subjects and Methods

### Subjects

All subjects gave written informed consent which was approved by the local ethics committee. We studied 19 subjects with type 2 diabetes, all Caucasian males between 50 and 65 years, who were recruited if they were non smokers, not taking dietary supplements, had never received gliclazide, antihypertensives, or angiotensin converting enzyme inhibitors, which have antioxidant or anti-inflammatory properties. Subjects were treated with diet alone (n = 5), metformin alone (n = 3), sulphonylureas alone (n = 4), metformin and sulphonylureas in combination (n = 3), and insulin alone or in combination with metformin (n = 4). Thirteen of the 19 subjects were taking an HMG CoA reductase inhibitor ('statin'). The volunteers were randomised into two groups, taking either 1200 IU α-tocopherol/d (n = 10) or matching placebo (n = 9) for 4 weeks. Compliance was monitored by pill count and plasma α-tocopherol concentrations. Table 1 summarises the clinical features of the two groups.

**Table 1:** Clinical and biochemical data groups in type 2 diabetes treated with 1200 IU α-tocopherol/day or placebo for 4 weeks

	α-tocopherol Group (n = 10)		Placebo Group (n = 9)	
	
	Day 0	Day 29	Day 0	Day 29
Age (Y)	62.7 (1.81)	__	61.9 (1.92)	__
Diabetes duration	11.1 (2.52)	__	2.6 (0.4)^a^	__
BMI	29.9 (1.2)	__	29.4 (1.3)	__
WHR	0.92 (0.026)	__	0.94 (0.021)	__
Fasting plasma glucose (mmol/L)	10.7 (0.94)	10.2 (0.86)	7.6 (0.39)^a^	7.7 (0.41)^b^
Fasting plasma insulin (mU/L)	49.6 (6.67)	56.2 (9.91)	55.5 (7.81)	54.5 (6.87)
Post-GTT plasma glucose (mmol/L)	20.2 (1.25)^c^	19.1 (1.26)^c^	15.1 (0.94)^c^	15.0 (1.32)^c^
Post-GTT plasma insulin (mU/L)	135 (37.1)^d^	124 (31.9)^d^	218 (47.7)^c^	261 (87.0)^d^
HbA1c (%)	8.4 (0.6)	__	7.2 (0.3)	__
Plasma α-tocopherol (μmol/L)	28.9 (1.82)	66.5 (7.26)^e^	24.4 (1.71)	25.0 (1.78)
8-oxoquanine fluorescence (MFI)	462 (28.0)	456 (19.0)	506 (27.1)	495 (15.2)

Data shown are means (SE)

^a ^p < 0.01 placebo v α-tocopherol; ^b ^p < 0.05 placebo v α-tocopherol;

^c ^p < 0.01 Fasting v post-GTT; ^d ^p < 0.05 Fasting v post-GTT

^e ^p < 0.01 Day 0 v Day 29

### Materials

On day 0 and day 29 of the study fasting blood samples were collected into vacutainer CPT tubes (Becton Dickinson, Oxford, UK). Volunteers were given a standard oral 75 g glucose tolerance test (GTT), and a further blood sample taken after 2 h. Mononuclear cells were separated by centrifugation. Oxidative DNA damage was assessed by measuring 8-oxoguanine (8-OG) using a Biotrin OxyDNA test kit (Biotrin International, Dublin, Ireland), as we have previously described [5]. In brief, 1 × 10^6 ^mononuclear cells were incubated with 1% paraformaldeyde for 15 min on ice, washed once with PBS, resuspended with 70% ethanol and kept at -20°C until analysed. Cells were washed with PBS then incubated with blocking buffer at 37°C for 1 h, washed twice, then incubated with FITC-labelled 8-OG probe for 1 h. The cells were washed twice and analysed by flow cytometry. Plasma insulin was measured using a human insulin-specific (no cross-reactivity with proinsulin) ELISA (Dako Cytomation, Ely, UK) and glucose by the glucose oxidase method. Plasma α-tocopherol was measured by HPLC as previously described [3].

Data are expressed as mean and one standard error (SE) or 95% confidence intervals (CI). Differences between groups were analysed by paired or unpaired two-tailed t-tests and significance taken as p < 0.05.

## Results

Both subject groups were matched for age, BMI and plasma insulin. However, by chance, the subjects allocated to the α-tocopherol treatment group had a significantly longer duration of diabetes (p < 0.01) and a higher fasting plasma glucose concentration than the placebo treatment group (p < 0.01).

### Baseline data

At the start of the study no differences were apparent in DNA damage, as assessed by 8-OG mean fluorescence intensity (MFI), in the mononuclear cells of the two groups (Table 1).

### Day 0, post GTT (GTT1)

**Figure 1:**
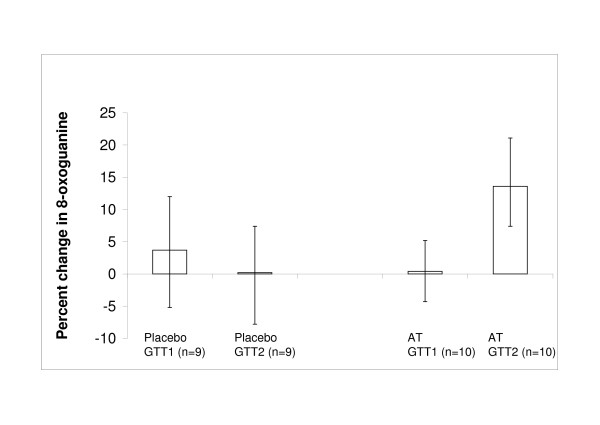
**Change in oxidative DNA damage, assessed by 8-oxoguanine fluorescence, in mononuclear cells isolated from type 2 diabetes patients before (GTT1) and after (GTT2) taking 1200 IU/day α-tocopherol or matched placebo for 4 weeks, following a glucose tolerance test**. Data shown are means; error bars represent 95% confidence intervals. AT: α-tocopherol, GTT: Glucose Tolerance Test

No significant change in the level of DNA damage was detected following a GTT in the mononuclear cells of either group (fig 1).

### Day 29, fasting sample

Following the 4 week intervention, plasma α-tocopherol was significantly increased (p < 0.01) in the α-tocopherol supplemented group but there was no significant change in mononuclear cell DNA damage in either treatment group (Table 1).

### Day 29, post GTT (GTT2)

There was a significant increase in DNA damage, assessed as a 13.6% (95% CI: 6.3–20.9) increase in 8-OG fluorescence, in the mononuclear cells from the α-tocopherol supplemented group following GTT (Fig 1).

A correlation (R = 0.649, p = 0.045), using simple linear regression, was observed between duration of diabetes and the percent change in 8-OG florescence following a GTT in the α-tocopherol supplemented group on day 29.

## Discussion

This study shows that after a relatively high dose of α-tocopherol for 4 weeks there was no change in oxidative DNA damage in mononuclear cells from subjects with type 2 diabetes, as we have reported previously [3]. However, following a glucose load with increased oxidative stress [2] the level of oxidative DNA damage increased significantly in α-tocopherol-supplemented type 2 diabetes patients which correlated with the duration of diabetes. This increase in oxidative DNA damage was not apparent in the placebo group. These data suggest that the high dose α-tocopherol treatment has primed a damaging response to acute hyperglycaemia in type 2 diabetes and may be related to the duration of the disease.

The ability of α-tocopherol to act as a pro-oxidant and increase peroxidation of lipids has been long known *in vitro *[12,13] and increased DNA damage, attributed to α-tocopherol, in cultured cells has been described following an insult capable of generating reactive oxygen species (ROS) [14,15]. Upon encountering ROS, α-tocopherol within lipid becomes oxidised forming its own radical, which requires co-antioxidants (e.g ascorbic acid) in order for the α-tocopherol to be regenerated. If the tocopherol radical is not eliminated there is an increase in lipid peroxidation, a process known as tocopherol-mediated peroxidation (TMP) [16]. These peroxidised lipids can produce a range of ROS which are able to damage DNA. H_2_O_2_, generated by TMP, while not damaging to DNA directly, is able to cross membranes and can react with transition metals (Fe, Cu) associated with DNA to generate hydroxyl radicals (^•^OH), by the Fenton reaction, to cause damage to DNA [17,18]. In addition Cu,Zn-Superoxide dismutase has been reported to release free copper when it is oxidatively damaged [19,20] which could lead to increased generation of ^•^OH.

Alpha-tocopherol has been reported to reduce γ-tocopherol concentrations in blood plasma [21] which could have a disadvantageous effect. Gamma-tocopherol is a potent scavenger of reactive nitrile species (RNS) such as nitric oxide (^•^NO) and peroxynitrate (ONOO-) [22], which may also damage biomolecules such as DNA. Peroxynitrate can also oxidise tetrahydrobioptrein (BH4), a co-factor of nitric oxide synthase (NOS) which causes uncoupling of NOS, resulting in the generation of superoxide (^•^O_2_) instead of nitric oxide (^•^NO) [23].

The timing of ingestion of supplements relative to meal times has been shown to effect markers of inflammation and may have an effect on oxidative stress [24], but the effects were seen following a single dose of vitamin E, whilst subjects on the current study, although only instructed to take supplements daily, had sustained elevated plasma levels of α-tocopherol. Other factors which may affect the outcomes of vitamin E supplementation have been discussed in a recent review [25].

High α-tocopherol intakes, at least without co-supplementation with other antioxidants such as vitamin C, which can reduce the α-tocopherol-induced lipid peroxidation observed *in vitro *[26], may result in amplification of ROS generated in response to an increase in oxidative stress and increased RNS due to suppression of γ-tocopherol bioavailability. High dose vitamin C, used in EDTA chelation therapy, has been shown to have a pro-oxidant effect [27] and ceruloplasmin, a copper containing metalloenzyme, has been suggested to have a pro-oxidant effect in conditions of increased oxidative stress, such as diabetes, by the disruption of copper binding [28]. A recent report [29] has suggested that the optimal serum concentration of α-tocopherol to reduce mortality from cardiovascular disease and cancer is 30–33 μmol/L. The mean plasma α-tocopherol concentration achieved in the supplemented group in this study was 66.5 μmol/L which may be high enough to be detrimental rather than beneficial.

In a recent review of oxidative stress and antioxidant use, Johansen *et al *[30] point out that most clinical trials conducted to date were not designed to specifically assess the effects of antioxidant use in diabetic patients, who experience a high level of oxidative stress, and that endpoints measured did not include specific markers of oxidative stress. However, in the current study we measured levels of 8-oxoguanine, a specific marker of oxidative DNA damage formed during free radical damage to DNA.

## Limitations

This study does have some limitations. First, the patient numbers are too low to form definitive conclusions. Second, the patients are taking different pharmacological treatments, although only subjects who had never received gliclazide, antihypertensives or ACE inhibitors, which have anti-oxidant or anti-inflammatory properties, were included in this study. Thirdly, there is a mismatch in duration of diabetes and plasma glucose between the two groups, which could affect the results. However HbA1c was not significantly different between each group and neither group differed significantly in their fasting levels of mononuclear cell oxidative damage either at the beginning or end of the study, nor showed any significant change following a GTT prior to supplementation.

## Conclusion

This report is to our knowledge the first to show a pro-oxidant action of α-tocopherol associated with increased DNA damage in patients with type 2 diabetes. Although this is a small sample these preliminary findings, given the observed correlation between duration of diabetes and increase in oxidative damage, suggest the possibility that high dose vitamin E is potentially more damaging in patients with longer duration of disease and needs further investigation. This data may be useful in interpreting negative vascular outcomes in large α-tocopherol intervention trials in subjects with increased basal levels of oxidative stress such as type 2 diabetes or atherosclerosis.

## Abbreviations

8-OG: 8-oxoguanine

AT: α-tocopherol

GTT: Glucose tolerance test

ROS: Reactive oxygen species

RNS: Reactive nitrile species

TMP: tocopherol-mediated peroxidation

## Competing interests

The author(s) declare that they have no competing interests.

## Authors' contributions

MSW measured 8-oxoguanine fluorescence and drafted the manuscript

MJS and DAH conceived and planned the study

SS measured serum α-tocopherol levels

JCH performed immunoassays

## References

[B1] Jay D, Hitomi H, Griendling KK (2006). Oxidative stress and diabetic cardiovascular complications. Free Radical Biology and Medicine.

[B2] Sampson MJ, Gopaul N, Davies IR, Hughes DA, Carrier MJ (2002). Plasma F2 Isoprostanes: Direct evidence of increased free radical damage during acute hyperglycemia in type 2 diabetes. Diabetes Care.

[B3] M.J S, S A, S R, G W, I.R D, DA H, S S (2001). Increased DNA oxidative susceptibility without increased plasma LDL oxidizability in Type II diabetes: effects of a-tocopherol supplementation. Clinical Science.

[B4] Dandona P, Thusu K, Cook S, Snyder B, Makowski J, Armstrong D, Nicotera T (1996). Oxidative damage to DNA in diabetes mellitus. The Lancet.

[B5] Sampson MJ, Winterbone MS, Hughes JC, Dozio N, Hughes DA (2006). Monocyte Telomere Shortening and Oxidative DNA Damage in Type 2 Diabetes. Diabetes Care.

[B6] The HOPE and HOPE-TOO Trial Investigators (2005). Effects of Long-term Vitamin E Supplementation on Cardiovascular Events and Cancer: A Randomized Controlled Trial. JAMA.

[B7] Heart Protection Study Collaborative G (2002). MRC/BHF Heart Protection Study of antioxidant vitamin supplementation in 20 536 high-risk individuals: a randomised placebo-controlled trial. The Lancet.

[B8] Miller ER, Pastor-Barriuso R, Dalal D, Riemersma RA, Appel LJ, Guallar E (2005). Meta-Analysis: High-Dosage Vitamin E Supplementation May Increase All-Cause Mortality. Ann Intern Med.

[B9] Steinberg D, Witztum JL (2002). Is the Oxidative Modification Hypothesis Relevant to Human Atherosclerosis?: Do the Antioxidant Trials Conducted to Date Refute the Hypothesis?. Circulation.

[B10] Halliwell B (2000). The antioxidant paradox. The Lancet.

[B11] Sardas S, Yilmaz M, Oztok U, Cakir N, Karakaya AE (2001). Assessment of DNA strand breakage by comet assay in diabetic patients and the role of antioxidant supplementation. Mutation Research/Genetic Toxicology and Environmental Mutagenesis.

[B12] Bowry VM, Igold S, Stocker R (1992). Vitamin E in human low-density lipoprotein. When and how this antioxidant becomes a pro-oxidant.. Biochemical Journal.

[B13] Santanam N, Parthasarathy S (1995). Paradoxical actions of antioxidants in the oxidation of low density lipoprotein by peroxidases.. J Clin Invest.

[B14] Nocentini S, Guggiari M, Rouillard D, Surgis S (2001). Exacerbating Effect of Vitamin E Supplementation on DNA Damage Induced in Cultured Human Normal Fibroblasts by UVA Radiation.. Photochemistry and Photobiology.

[B15] Blasiak J, Gloc E, Wozniak K, Mlynarski W, Stolarska M, Skorski T, Majsterek I (2002). Genotoxicity of idarubicin and its modulation by vitamins C and E and amifostine. Chemico-Biological Interactions.

[B16] Stocker R (1999). The ambivalence of vitamin E in atherogenesis. Trends in Biochemical Sciences.

[B17] Yamashita N, Murata M, Inoue S, Burkitt MJ, Milne L, Kawanishi S (1998). Tocopherol Induces Oxidative Damage to DNA in the Presence of Copper(II) Ions. Chem Res Toxicol.

[B18] Henle ES, Luo Y, Gassmann W, Linn S (1996). Oxidative Damage to DNA Constituents by Iron-mediated Fenton Reactions. THE DEOXYGUANOSINE FAMILY. J Biol Chem.

[B19] Sato K, Akaike T, Kohno M, Ando M, Maeda H (1992). Hydroxyl radical production by H2O2 plus Cu,Zn-superoxide dismutase reflects the activity of free copper released from the oxidatively damaged enzyme. J Biol Chem.

[B20] Yoon SJ, Koh YH, Floyd RA, Park JW (2000). Copper, zinc superoxide dismutase enhances DNA damage and mutagenicity induced by cysteine/iron. Mutation Research/Fundamental and Molecular Mechanisms of Mutagenesis.

[B21] Wolf G (2006). How an Increased Intake of Alpha-Tocopherol Can Suppress the Bioavailability of Gamma-Tocopherol. Nutrition Reviews.

[B22] Jiang Q, Christen S, Shigenaga MK, Ames BN (2001). {gamma}-Tocopherol, the major form of vitamin E in the US diet, deserves more attention. Am J Clin Nutr.

[B23] Laursen JB, Somers M, Kurz S, McCann L, Warnholtz A, Freeman BA, Tarpey M, Fukai T, Harrison DG (2001). Endothelial Regulation of Vasomotion in ApoE-Deficient Mice : Implications for Interactions Between Peroxynitrite and Tetrahydrobiopterin. Circulation.

[B24] Carroll MF, Schade DS (2003). Timing of Antioxidant Vitamin Ingestion Alters Postprandial Proatherogenic Serum Markers. Circulation.

[B25] Robinson I, de Serna DG, Gutierrez A, Schade DS (2007). Vitamin E in Humans: An Explanation of Clincal Trial Failure.. Endocrine Practice.

[B26] Bowry VW, Stocker R (1993). Tocopherol-mediated peroxidation. The prooxidant effect of vitamin E on the radical-initiated oxidation of human low-density lipoprotein. Journal of the American Chemical Society.

[B27] Hininger I, Waters R, Osman M, Garrel C, Fernholz K, Roussel AM, Anderson RA (2005). Acute prooxidant effects of vitamin C in EDTA chelation therapy and long-term antioxidant benefits of therapy. Free Radical Biology and Medicine.

[B28] Shukla N, Maher J, Masters J, Angelini GD, Jeremy JY (2006). Does oxidative stress change ceruloplasmin from a protective to a vasculopathic factor?. Atherosclerosis.

[B29] Wright ME, Lawson KA, Weinstein SJ, Pietinen P, Taylor PR, Virtamo J, Albanes D (2006). Higher baseline serum concentrations of vitamin E are associated with lower total and cause-specific mortality in the Alpha-Tocopherol, Beta-Carotene Cancer Prevention Study. Am J Clin Nutr.

[B30] Johansen JS, Harris AK, Rychly DJ, Adviye E (2005). Oxidative stress and the use of antioxidants in diabetes: Linking basic science to clinical practice. Cardiovascular Diabetology.

